# Hybrid Harris hawks optimization with cuckoo search for drug design and discovery in chemoinformatics

**DOI:** 10.1038/s41598-020-71502-z

**Published:** 2020-09-02

**Authors:** Essam H. Houssein, Mosa E. Hosney, Mohamed Elhoseny, Diego Oliva, Waleed M. Mohamed, M. Hassaballah

**Affiliations:** 1 Faculty of Computers and Information, Minia University, Minia, Egypt; 2 Faculty of Computers and Information, Luxor University, Luxor, Egypt; 3 Faculty of Computers and Information, Mansoura University, Mansoura, Egypt; 4 Depto. de Ciencias Computacionales, Universidad de Guadalajara, CUCEI, Guadalajara, Jal Mexico; 5 IN3 - Computer Science Department, Universitat Oberta de Catalunya, Castelldefels, Spain; 6 Computer Science Department, Faculty of Computers and Information, South Valley University, Qena, Egypt

**Keywords:** Chemical tools, Cheminformatics

## Abstract

One of the major drawbacks of cheminformatics is a large amount of information present in the datasets. In the majority of cases, this information contains redundant instances that affect the analysis of similarity measurements with respect to drug design and discovery. Therefore, using classical methods such as the protein bank database and quantum mechanical calculations are insufficient owing to the dimensionality of search spaces. In this paper, we introduce a hybrid metaheuristic algorithm called CHHO–CS, which combines Harris hawks optimizer (HHO) with two operators: cuckoo search (CS) and chaotic maps. The role of CS is to control the main position vectors of the HHO algorithm to maintain the balance between exploitation and exploration phases, while the chaotic maps are used to update the control energy parameters to avoid falling into local optimum and premature convergence. Feature selection (FS) is a tool that permits to reduce the dimensionality of the dataset by removing redundant and non desired information, then FS is very helpful in cheminformatics. FS methods employ a classifier that permits to identify the best subset of features. The support vector machines (SVMs) are then used by the proposed CHHO–CS as an objective function for the classification process in FS. The CHHO–CS-SVM is tested in the selection of appropriate chemical descriptors and compound activities. Various datasets are used to validate the efficiency of the proposed CHHO–CS-SVM approach including ten from the UCI machine learning repository. Additionally, two chemical datasets (i.e., quantitative structure-activity relation biodegradation and monoamine oxidase) were utilized for selecting the most significant chemical descriptors and chemical compounds activities. The extensive experimental and statistical analyses exhibit that the suggested CHHO–CS method accomplished much-preferred trade-off solutions over the competitor algorithms including the HHO, CS, particle swarm optimization, moth-flame optimization, grey wolf optimizer, Salp swarm algorithm, and sine–cosine algorithm surfaced in the literature. The experimental results proved that the complexity associated with cheminformatics can be handled using chaotic maps and hybridizing the meta-heuristic methods.

## Introduction

The prediction and analysis of molecules are essential tasks in cheminformatics, which use methods from mathematics and computer science to enhance their performance. The implementation of these methods depends on databases. The processes that generate most of the affectations are the storage and retrieval of molecular structures and properties (e.g., pharmacogenomics data). Typically, the behavior of the compounds can be investigated using molecular analysis. The molecular analysis helps to develop and test molecules for decreasing the effects of specific diseases^1^. One drawback associated with cheminformatics is the exponential increment of the search space owing to features in the dataset^2^. However, cheminformatics is still being widely used in drug design, where the protein structures are estimated and the interactions of molecules and biological targets can be determined by considering the basis of the cellular processes^1^.

A drug is an organic molecule that can inhibit the effects of a disease. The main points for drug design and discovery are: (1) structure optimization^3^, (2) establishment of the quantitative structure-activity relationship (QSAR)^4^, and (3) docking of the ligand into a receptor denovo design of ligands^5^. Thus, drug design and discovery aim to develop new medicines based on the knowledge about a biological target^6^. The features contained in the datasets are essential for cheminformatics, but due to the big amount of generated information, it results in complicated to handle them in most of the cases^7^.

Generally speaking, feature selection (FS) is an important preprocessing step for performance enhancement in data mining. FS is especially used for classification and regression problems. FS approaches are widely used to eliminate the irrelevant and redundant features from the original dataset, therefore, the dimensionality of the dataset is reduced^8^. As was mentioned cheminformatic datasets are huge and the use of FS is mandatory in order to identify the best subset of information. Typically, the FS approaches can be divided into wrapper and filter methods^9^. The wrapper-based approaches often cope with the filters, because the proposed subset of features is directly assessed using feedback from the learning algorithm as to its accuracy^10,11^. In the wrapper techniques, the option of using machine learning algorithms is wide open, then it is possible to find implementations of the most popular algorithms including support vector machines (SVMs) and K-nearest neighbor (KNN), among others. Nevertheless, in order to find an efficient FS technique, researchers have put significant efforts, particularly those working with metaheuristic algorithms (MAs). In this regard, a wide spectrum of MAs are either used alone^12^ or with others to form hybrid methods^13^ for efficient results, since a comprehensive list can be easily found in this review^14^.

Due to the success of MAs in solving complex problems^15^, they can be employed in cheminformatics. Harris hawks optimization (HHO) is a recent method introduced in^16^. Apart from its novelty, HHO is a powerful optimization tool that is robust, exhibits smooth transitions between exploration and exploitation, and provides competitive results to complex problems^17^. However, there is no perfect MA, and HHO has some disadvantages. In HHO, exploration, and exploitation are unbalanced and it has premature convergence when the problems are highly multimodal^18^. In this context, the cuckoo search (CS) algorithm is inspired by the breeding behavior of the cuckoo birds. It has been introduced as an alternative method for global optimization^19^. Since its publication, CS has been widely used by the scientific community^20–22^. In addition, CS is applied for secondary protein structure prediction^23^. Generally, the advantages of CS are that it ensures global convergence and maintains a well balance between exploration and exploitation^24^. The use of L$\acute{e}$vy flights in CS permits them to perform a successful global search, which is reflected in their capabilities to obtain space using sub-optimal solutions. However, chaos is part of the nonlinear dynamic systems. Chaos is described as a behavior of complex systems, where small, random, and unpredictable changes can be observed over time with respect to the initial conditions. The concepts of chaos are helpful in optimization because they help to generate accurate solutions. Chaos is commonly used instead of random distributions to improve MA performance^25^. The inclusion of chaotic maps in optimization methods increases the diversity of solutions by avoiding local solutions and speeding up the convergence.

In the basic HHO, the control energy parameter *E*, as well as the position vectors, called $X_{rand}$ and $X_{rabbit}$ plays the main role in avoiding the local optima and balancing the exploitation and exploration. Therefore, in this study, we introduce a hybrid method that combines the benefits of HHO with those of CS and chaotic maps (C); this algorithm can be referred to as CHHO–CS. The concept of the CHHO–CS is to enhance the search process of HHO to obtain near-optimal solutions. To be specific, a new formulation of the initial escape energy $E_{0}$, escaping energy factor *E* and the initialization of solutions with chaotic maps are presented. The inclusion of chaotic maps may avoid the local optima and accelerates the convergence. Additionally, in CHHO–CS method, CS is used to control the position vectors called $X_{rand}$ and $X_{rabbit}$ of the basic HHO. The objective (or fitness) function is then shared in the entire optimization process. It means that the CS works with the same objective function used by HHO. Finally, the CHHO–CS is combined with the support vector machine (SVM) to select the appropriate chemical descriptors (features) and compounds activities. In addition, this study investigates the influence of the chaotic map with respect to the cheminformatics problems. Several experiments and comparisons have been conducted with respect to different versions to select the version which provides the most accurate solutions. Furthermore, twelve datasets are used to evaluate the efficiency of CHHO–CS compared to seven well-known metaheuristic algorithms, including: HHO^16^, CS^19^, particle swarm optimization (PSO)^26^, moth-flame optimization (MFO)^27^, grey wolf optimizer (GWO)^28^, salp swarm algorithm (SSA)^29^, and sine–cosine algorithm (SCA)^30^. The CHHO–CS method achieves the best results of classification accuracy and the number of selected features when compared with the remaining competitor algorithms. The major contributions of this work are as follows:

1. A new CHHO–CS method is proposed based on combining HHO with the benefits of CS and chaotic maps. CS and chaotic maps (C) are used to enhance the limitations of the original HHO.
2. The SVM classifier is utilized in the CHHO–CS to select the chemical descriptors and chemical compound activities.
3. Several experiments are conducted on various datasets to confirm the superiority of the proposed CHHO–CS method in combination with SVM compared with other metaheuristic algorithms.

The rest of this paper is structured as follows. Literature review is presented in “Related work” section. “Materials and methods” section introduces the necessary material and methods used in the study, such as QSAR, SVM, HHO, the theory of Cuckoo search (CS) algorithm, and the chaotic maps. Meanwhile, “The proposed CHHO–CS” section explains the pre-processing process and introduces the proposed CHHO–CS method. The experimental result and discussion are presented in “Results” section. Finally, the conclusion of the paper is provided in “conclusion” section.

## Related work

A previously conducted study has investigated drug design and discovery, exhibiting differences in efficiency^31^. The available tools used to identify chemical compounds which are known as computer-aided drug design (CADD) allows the reduction of different risks associated with the subsequent rejection of lead compounds. CADD has an important role and exhibits high success rates for the identification of the hit compounds^32^.

The CADD methodology has two related concepts: ligand/hit optimization and ligand/hit identification. Methods hitting identification/optimization are based on the efficiency of the virtual screening techniques used to achieve the target binding sites. They are known to dock huge libraries for small molecules including chemical information or ZINC database, to identify the compounds based on the pharmacophore modeling tools (docking) to predict the optimal medicines and proteins obtained using the information from the ligand. The Pymol software^33^ is useful in selecting the optimal ligand as the optimal drug, and the AutoDock software is employed to calculate the energy^5^. Thus, genetic algorithms (GAs) are applied in the AutoDock software and AutoDock Vina^34^. Also, in^35^, fuzzy systems have been introduced to address the optimization of the chemical product design. Another important method for drug design called QSAR is derived from CADD to extract the description of the correlation among different structures from a set of molecules and the response to the target^36^.

Drug design and discovery are the main aspects of cheminformatics^37^. Cheminformatics can be divided into two sub-processes. The first process considers three-dimensional information; this process is called encoding. The second process, which is called mapping, comprises building a model using machine learning (ML) techniques^38^. In the encoding process, the molecular structure is transformed based on the calculation of the descriptors^36^. Moreover, the mapping process aims to discover different mappings created between the feature vectors and their properties. In cheminformatics and drug discovery, the mapping can be performed using various machine learning^2,39^.

Chaotic maps are random-like deterministic methods that constitute dynamic systems. They have nonlinear distributions indicating that chaos is a simple deterministic dynamic system and a source of randomness. Chaos has random variables instead of chaotic variables and absolute searches can be performed with higher speeds when compared with stochastic search methods mainly based on probabilities. In a previous study^40^, chaotic maps have been considered to improve the performance of the whale optimization algorithm and balance the exploration and exploitation phases. Also, a grey wolf optimizer and flower pollination algorithm have been enhanced using ten chaotic maps to extract the parameters of the bio-impedance models^41^. Meanwhile, in^42^, the grasshopper optimization algorithm with chaos theory is employed to accelerate its global convergence and avoid local optimal. In^43^ the schema of the CS algorithm based on a chaotic map variable value is introduced.

In fact, the methodology of hybridizing MAs is widely used in different domains of optimization other than feature selection^44^. In this vein, combinations of different ML techniques and MAs (e.g., search strategies) have been applied in many fields with modifications and hybridization to benefit from one technique in uplifting search efficiency. For instance, the salp swarm algorithm combined with k-NN based on QSAR is an interesting alternative, which provides competitive solutions^45^. Also, Houssein et al.^37^ introduced a novel hybridization approach for drug design and discovery-based hybrid HHO and SVM. However, in this study, we applied hybridization to select the chemical descriptor and compound activities in cheminformatics. Particularly, this study proposes an alternative classification approach with respect to cheminformatics, termed as CHHO–CS-based SVM classifier, for selecting the chemical descriptor and chemical compound activities; the hybrid HHO and CS were enhanced based on the chaos (C) theory.

## Materials and methods

In this section, we briefly discus the QSAR model, the basics of SVM, the original HHO, the original CS, and the chaotic map theory.

### Quantitative structure-activity relationship

QSAR provides information based on the relation between the mathematical models associated with the biological activity and the chemical structures. QSAR is widely used because it can detect major characteristics of the chemical compounds. Therefore, it is not necessary to test and synthesize compounds. The inclusion of ML methods to study QSAR helps to predict whether the compound activity is similar to a drug-like activity in case of a specific disease or a chemical test. The compounds possess complex molecular structures, containing many attributes for their description. Some of the features include characterization and topological indices. Therefore, molecular descriptors are highly important in pharmaceutical sciences and chemistry^4^.

### Support vector machine

**Figure 1:**
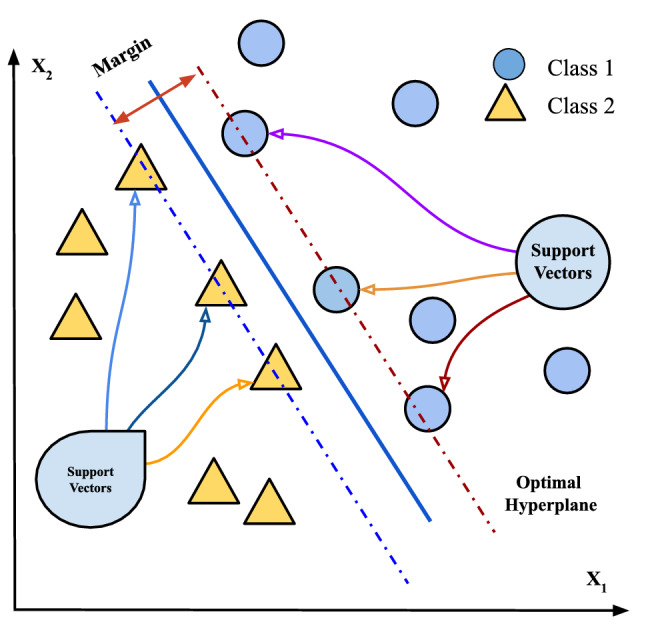
General structure of a decision boundary in SVMs classification.

SVM is an important supervised learning algorithm commonly used for classification^46^. SVM extracts different points from the data and maps them in a high-dimensional space using a nonlinear kernel function. SVM works by searching for the optimal solution for class splitting. The solution can be used to maximize the distance with respect to the nearest points defined as support vectors, and the result of SVM is a hyperplane. For obtaining optimal results, SVM has some parameters that have to be tuned. The *C* controls the interaction between smooth decision boundaries and the accurate classification of the training points. If the *C* has a significant value, more training points will be accurately obtained, indicating that more complex decision curves will be generated by attempting to fit in all the points. The different values of *C* for a dataset can be used to obtain a perfectly balanced curve and prevent over-fitting. $\Gamma$ is utilized to characterize the impact of single training. Low gamma implies that each point will have a considerable reach, whereas high gamma implies that each point has a close reach. The implementation of SVM has been extended to cheminformatics. In this work, steps of SVM are presented in Algorithm 1, and its graphical description is presented in Fig. 1. 
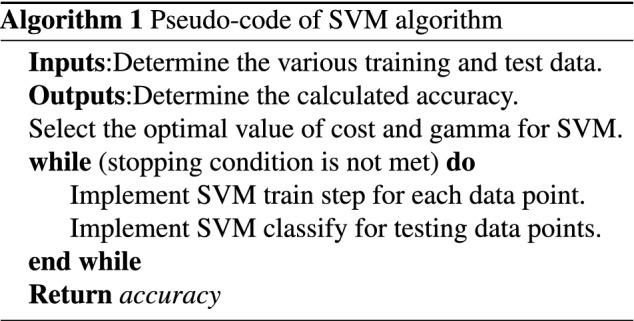

### Harris hawks optimization

HHO^16^ is a metaheuristic algorithm and is implemented as a competitive solution for complex problems. HHO is inspired by the attitude of Harris hawks, which are intelligent birds. This species possesses a mechanism that allows them to catch prey even when they are escaping. This process is modeled in the form of a mathematical expression, allowing its computational implementation. HHO is a stochastic algorithm that can explore complex search spaces to find optimal solutions. The basic steps of HHO can be obtained with respect to various states of energy. The exploration phase simulates the mechanism when Harris’s hawk cannot accurately track the prey. In such a case, the hawks take a break to track and locate new prey. Candidate solutions are the hawks in the HHO method, and the best solution in every step is prey. The hawks randomly perch at different positions and wait for their prey using two operators, which are selected on the basis of probability *q* as given by Eq. (1), where $q<0.5$ indicates that the hawks perch at the location of other population members and the prey (e.g., rabbit). If $q\ge 0.5$, the hawks are at random positions around the population range. For facilitating the understanding of HHO, a list of symbols used in this algorithm is defined as follows:

1. Vector of hawks position (search agents) $X_{i}$
2. Position of Rabbit (best agent) $X_{rabbit}$
3. Position of a random Hawk $X_{rand}$
4. Hawks average position $X_{m}$
5. Maximum number of iterations, swarm size, iteration counter *T*, *N*, *t*
6. Random numbers between (0, 1) $r_{1}$, $r_{2}$, $r_{3}$, $r_{4}$, $r_{5}$, *q*
7. Dimension, lower and upper bounds of variables *D*, *LB*, *UB*
8. Initial state of energy, escaping energy $E_{0}$, *E*

The exploration step is defined as:

1 $$\begin{aligned} \begin{aligned} X(t+1) = \left\{ \begin{array}{ll} X_{rand}(t)-r_{1}\left| X_{rand}(t)-2r_{2}X(t) \right| &{}\quad q\ge 0.5 \\ (X_{rabbit}(t)-X_{m}(t))-r_{3}(LB+r_{4}(UB-LB)) &{} \quad q<0.5 \end{array}\right. \end{aligned} \end{aligned}$$

The average location of the Hawks $X_m$ is represented by:

2 $$\begin{aligned} X_{m}(t)=\frac{1}{N}\sum _{i=1}^{N}X_{i}(t) \end{aligned}$$

where $X_{i}(t)$ shows the positions in the iteration for each Hawk *t* and *N* identifies the total number of Hawks. The average position can be obtained by using different methods, but this is the simplest rule. A good transition from exploration to exploitation is required, here a shift is expected between the different simulated exploitative behaviors based on the escaping energy factor *E* of the prey, which diminishes dramatically during the escaping behavior. The energy of the prey is computed by Eq. (3).

3 $$\begin{aligned} E=2E_{0} \left( 1-\frac{t}{T}\right) \end{aligned}$$

where *E*, $E_0$, and *T* represent the initial escape energy, the escape energy and the maximum number of iterations, respectively.

The soft besiege is an important step in HHO, it is shown if $r\ge 0.5$ and $|E|\ge 0.5$. In this scenario, the rabbit has all sufficient energy. When it occurs, the rabbit performs random misleading shifts to escape, but in the metaphor, it cannot. The besiege step is defined by the following rules:

4 $$\begin{aligned} X(t+1)= & {} \Delta X(t)-E\left| JX_{rabbit}(t)-X(t)\right| \end{aligned}$$

5 $$\begin{aligned} \Delta X(t)= & {} X_{rabbit}(t)-X(t) \end{aligned}$$

where $\Delta X(t)$ is the difference locations vector for all rabbits and for presently positions in the iteration *t*, and $J=2(1-r_{5})$ Is the rabbit’s spontaneous jumping ability throughout the escaping phase. The *J* value varies randomly in each iteration to represent the rabbit’s behavior. In the extreme siege stage when $r\ge 0.5$ and $|E|<0.5$, The prey is exhausted and has no escaping strength. The Harris hawks are hardly circling the trained prey, and they can make an assault of surprise. For this case, the current position is changed using:

6 $$\begin{aligned} X(t+1)=X_{rabbit}(t)-E \left| \Delta X(t) \right| \end{aligned}$$

Consider the behavior of hawks in real life, they will gradually choose the best dive for the prey if they want to capture specific prey in competitive situations. This is simulated by:

7 $$\begin{aligned} Y=X_{rabbit}(t)-E\left| JX_{rabbit}(t)-X(t)\right| \end{aligned}$$

The soft besiege presented in the previous Eq. (7) is performed in progressive rapid dives only if $|E|\ge 0.5$ but $r<0.5$. In this case, the rabbit has sufficient energy to escape and is applied for a soft siege before the attack comes as a surprise. The HHO models have different patterns of escape for a leap frog and prey movements. The Lévy flights (LF) are launched here to emulate the various movements of the Hawk and rabbit dives. Eq. (8) computes such patterns.

8 $$\begin{aligned} Z=Y+S\times LF(D) \end{aligned}$$

where *S* represents the random vector for size $1\times D$ and LF is for the levy flight function, using this Eq. (9):

9 $$\begin{aligned} LF(x)=0.01\times \frac{u\times \sigma }{\left| v \right| ^{\frac{1}{\beta }}}, \sigma = \left( \frac{\Gamma (1+\beta )\times sin \left( \frac{\pi \beta }{2}\right) }{\Gamma \left( \frac{1+\beta }{2}\right) \times  \beta \times 2^{\left( \frac{\beta -1}{2}\right) } } \right) ^{\frac{1}{\beta }} \end{aligned}$$

Here *u*, *v* are random values between (0, 1), $\beta$ is the default constant set to 1.5.

The final step in the process is to update positions of the hawks using:

10 $$\begin{aligned} X(t+1)=\left\{ \begin{array}{ll} Y &{}\quad if\; F(Y)<F(X(t)) \\ Z &{}\quad if \; F(Z)<F(X(t)) \\ \end{array}\right. \end{aligned}$$

where *Y* and *Z* are obtained using Eqs. (7) and (8).

During progressive fast dives, HHO is also hard-pressed, where it may happen if $|E|< 0.5$ and $r<0.5$. Here the strength of the rabbit to escape is not sufficient and the hard siege is suggested before the numerous surprise attacks are made to catch and kill the prey. In this step, Hawks seek to reduce the various distances between their prey and the average position. This operator is explained as follows:

11 $$\begin{aligned} X(t+1)=\left\{ \begin{array}{ll} Y &{}\quad if\; F(Y)<F(X(t)) \\ Z &{}\quad if\; F(Z)<F(X(t)) \\ \end{array}\right. \end{aligned}$$

The values of *Y* and *Z* are proposed by using new rules in Eqs. (12) and (13), where $X_{m}(t)$ is obtained using Eq. (2).

12 $$\begin{aligned} Y= & {} X_{rabbit}(t)-E\left| JX_{rabbit}(t)-X_{m}(t)\right| \end{aligned}$$

13 $$\begin{aligned} Z= & {} Y+S\times LF(D) \end{aligned}$$

### Cuckoo search

Fundamentally, Cuckoo Search (CS) is a metaheuristic algorithm used often for solving complex problems of optimization^19^. The cuckoo quest hypothesis is inspired by a bird known as the cuckoo. Cuckoos are interesting creatures not only because they can make beautiful sounds but also for their aggressive strategy of reproduction. In the nests of other host birds or animals, adult cuckoos lay their eggs. Cuckoo search is based on three main rules:

1. Growing cuckoo lays one egg at a time and dumps the egg in a nest selected randomly.
2. The best nest with high-quality eggs will be delivered to the next generation.
3. The number of host nests available is set and the host bird finds the egg laid by a cuckoo with a probability $\rho _{a} \in [0, 1]$.

The probability is based on these three rules such that the host bird can either throw away the egg or leave the nest and build a completely new nest. This statement may be approximated by a fraction $\rho _{a}$ of *n* nests that are replaced by new nests (with new random solutions). The pseudo-code of CS is shown in Algorithm 2. 
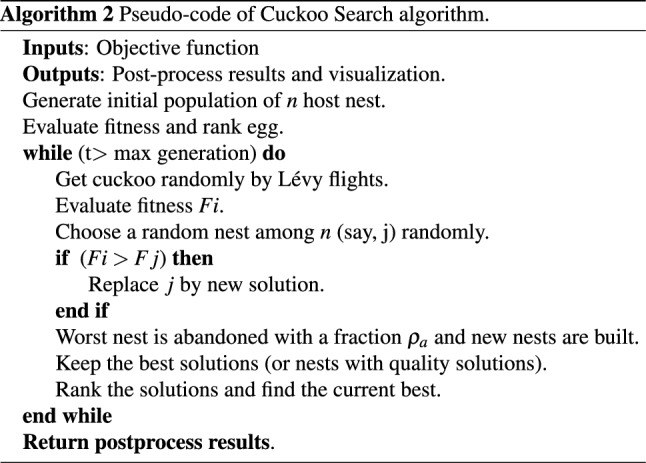


### Chaotic maps

The majority of MAs have been established based on stochastic rules. These rules primarily rely on certain randomness obtained using certain distributions of probabilities, which are often uniform or Gaussian. In principle, the replacement of this randomness with chaotic maps can be beneficial because of the significant dynamic properties associated with the behavior of chaos. This dynamic mixing is important to ensure that the solutions obtained using the algorithm are sufficiently diverse to enter any mode in the objective multimodal landscape. These approaches, which use chaotic maps, are called chaotic optimization instead of random distributions. The mixing properties of chaos will perform the search process at higher speeds than traditional searches based on the standard probability distributions^47^. One-dimensional non-invertible maps will be used to produce a set of variants of chaotic optimization algorithms to achieve this ability. Table 1 presents some of the prominent chaotic maps used in this study. In addition, chaotic maps are obliged to result in 0/1 based on the normalization concept.

The main task of chaotic maps is to avoid the local optima and speed up the convergence. Here, it is important to mention that the nature of chaotic maps could also increase the exploration due to the intrinsic randomness. It is necessary to properly select the best map that helps each algorithm for a specific problem. Another important point to be considered is that chaotic maps do not take decision about the exploration and exploitation of the algorithms. However, along with the iterations, the chaotic values generated by the maps permit to change the degree of exploration or exploitation of the search space.

**Table 1:** Details of chaotic maps applied on CHHO–CS.

No.	Map name	Ref.	Map equation	Notes
M1	Tent	^48^	$${{x}_{k+1}}=\left\{ \begin{array}{l} \frac{{x}_{k}}{0.7},{{x}_{k}}\prec 0.7 \\ \frac{10}{3}(1-{{x}_{k}}),{{x}_{k}}\ge 0.7 \\ \end{array} \right. $$	–
M2	Logistic	^49^	${{x}_{k+1}}=a{{x}_{k}}(1-{{x}_{k}})$	${{x}_{o}}\in (0,1)$ for *kth* chaotic number
M3	Sinusoidal	^49^	${{x}_{k+1}}=ax_{k}^{2}\sin (\pi {{x}_{k}})$	$\mu$ is a parameter between 0.9 and 1.08
M4	Singer	^50^	${{x}_{k+1}}=\mu (7.86{{x}_{k}}-23.31x_{k}^{2}+28.75x_{k}^{3}-13.3x_{k}^{4})$	–
M5	Sine	^51^	${{x}_{k+1}}=\frac{a}{4}\,\,\sin (\pi {{x}_{k}})$	$0\prec a\prec 4$
M6	Chebyshev	^52^	${{x}_{k+1}}=\cos (k\,{{\cos }^{-1}}({{x}_{k}}))$	–
M7	Circle	^53^	${{x}_{k+1}}={{x}_{k}}+b-(\frac{a}{2\pi })\sin (2\pi {{x}_{k}})\bmod$	a = 0.5 and b = 0.2, it generates chaotic sequence in (0, 1)
M8	Iterative	^54^	${{x}_{k+1}}=\sin (\frac{a\pi }{{{x}_{k}}})$	$a\in (0, 1)$
M9	Gauss/Mouse	^55^	$${{x}_{k+1}}=\left\{ \begin{array}{l} 0\,{{x}_{k}}=0 \\ \frac{1}{{{x}_{k}}\bmod (1)},otherwise \\ \end{array} \right. $$	Generates chaotic sequences in (0, 1)
			$$\frac{1}{{{x}_{k}}\bmod (1)}=\frac{1}{{{x}_{k}}}-\left[ \frac{1}{{{x}_{k}}} \right] $$	
M10	Piecewise	^56^	$${{x}_{k+1}}=\left\{ \begin{array}{l} \frac{{x}_{k}}{P},0\le {{x}_{k}}\prec P \\ \frac{{x}_{k}-P}{0.5-P}\,P\le {{x}_{k}}\prec 0.5 \\ \frac{1-P-{{x}_{k}}}{o.5-P},0.5\le {{x}_{k}}\prec 1-P \\ \frac{1-{{x}_{k}}}{P},1-P\le {{x}_{k}}\prec 1 \\ \end{array} \right. $$	The control parameter $P\in (0,0.5)$ and $x\in (0,1)$ and $P\ne 0$

## The proposed CHHO–CS

In this section, the proposed CHHO–CS is explained in detail, which is used to improve the search-efficiency of basic HHO. Typically, HHO has the characteristics of acceptable convergence speed and a simple structure. However, for some complex optimization problems, HHO may fail to maintain the balance between exploration and exploitation and fall into a local optimum. Especially in the face of high dimension functions and multi-modal problems, the shortcomings of HHO are more obvious. The optimization power of the basic HHO depends on the optimal solution^57^. In this paper, we introduced two strategies (Chaotic maps, and CS) to enhance the performance of the basic HHO.

**Figure 2:**
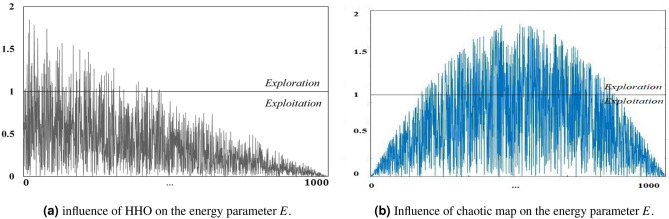
Influence of proper selection of energy parameter *E*.

The following points are worthwhile:

- Chaotic maps influence: applying chaos theory to the random search process of MAs significantly enhances the effect of random search. Based on the randomness of chaotic local search, MAs can avoid falling into local optimum and premature convergence. In the basic HHO algorithm, the transition from global exploration to local exploitation is realized according to Eq. (3). As a result, the algorithm will easily fall into a local optimum. Hence, in the CHHO–CS algorithm, a new formulation of initial escape energy $E_{0}$ and escaping energy factor *E* with chaotic maps are employed as demonstrated in Algorithm 3. Figure 2 shows the influence of a chaotic map on the energy parameter *E* obtained by the proposed method versus the basic HHO. Notably, the curve in the left-side linearly decreasing versus the proposed non-linear energy parameter defined by the new formulation of *E*, which clearly focuses on providing the search direction towards the middle of the search process to infuse enough diversity in population during the exploitation phase.
- CS method influence: in the basic HHO, the position vectors $X_{rand}$ and $X_{rabbit}$ are responsible for the exploration step defined by Eq. (1), which plays a vital role in balancing the exploitation and exploration. More significant values of position vectors expedite global exploration, while a smaller value expedites exploitation. Hence, an appropriate selection of $X_{rand}$ and $X_{rabbit}$ should be made, so that a stable balance between global exploration and local exploitation can be established^58^. Accordingly, in the CHHO–CS algorithm, we borrow the merits CS method to control the position vectors of HHO. At the end of each iteration *T*, CS trying to find the better solution (if better solution found then update $X_{rabbit}$ and $X_{rand}$; otherwise left obtained values by HHO unchanged). Consequently, CS will determine the fitness value of the new solution, if it is better than the fitness value of the obtained from HHO, then the new solutions will be set; otherwise the old remains unchanged.

**Figure 3:**
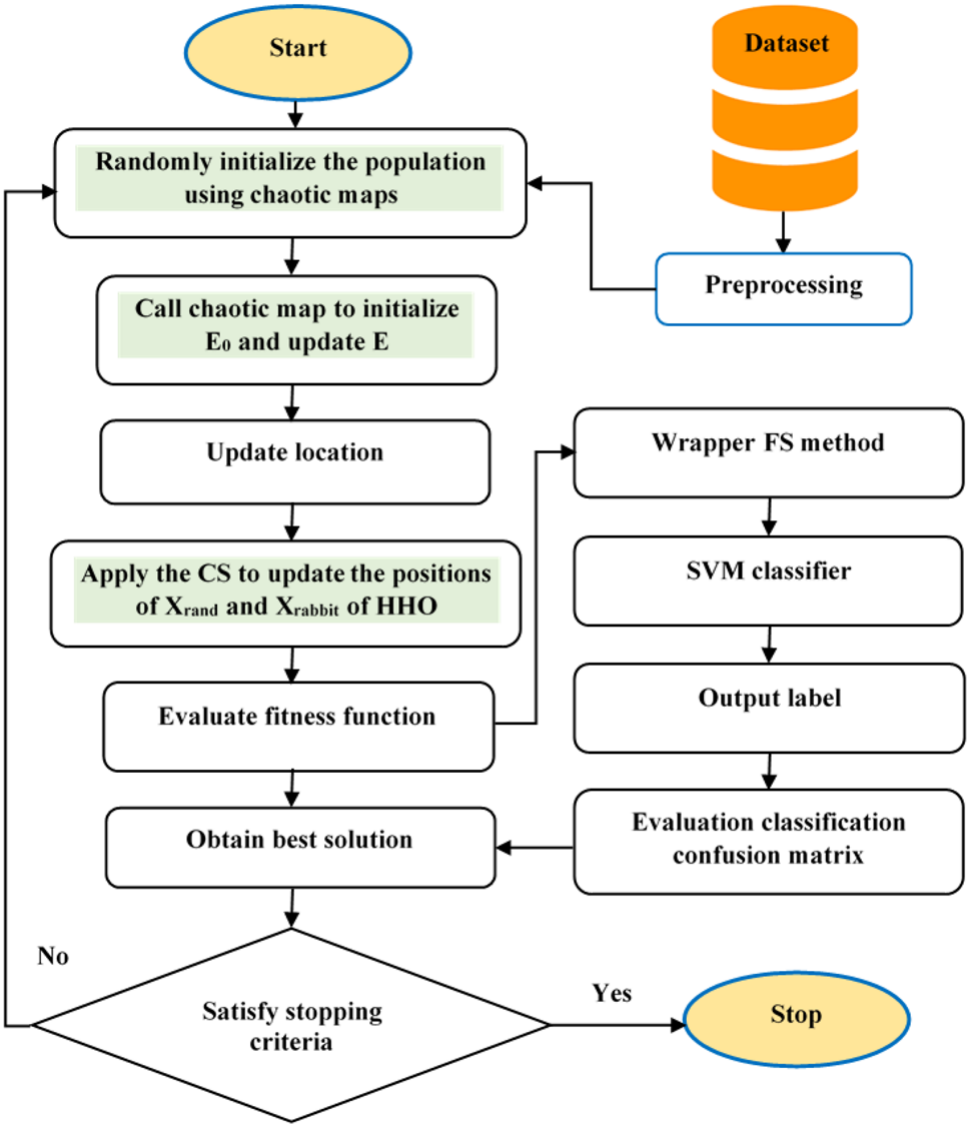
General flowchart of the proposed CHHO–CS method.

To be specific, the steps of the CHHO–CS algorithm are executed as; chaotic maps are employed to avoid falling into local optimum and premature convergence. Moreover, a balancing between exploration and exploitation is performed by CS. Then, SVM is used for classification purposes. The flowchart of the proposed CHHO–CS method is represented in Fig. 3. The pseudo-code of the proposed CHHO–CS method is illustrated in Algorithm 3. Here is important to mention that for SVM and feature selection, in the CHHO–CS each solution of the population is encoded as a set of indexes that correspond to the rows of the dataset. For example, if a dataset has 100 rows a possible candidate solution in the population for five dimensions could be [10, 20, 25, 50, 80], such values are rows with the features to be evaluated in the SVM. The location vector in the soft and hard besiege with progressive rapid dives in HHO is updated as follows:

14 $$\begin{aligned} X(t+1)=\left\{ \begin{array}{ll} Y &{} \quad if\; LF(fobj(D,G,Y))<LF(fobj(D,G,X((t))*X((t) \\ \\ Z &{}\quad if\; LF(fobj(D,G,Z))<LF(fobj(D,G,X((t))*X((t) \end{array}\right. \end{aligned}$$

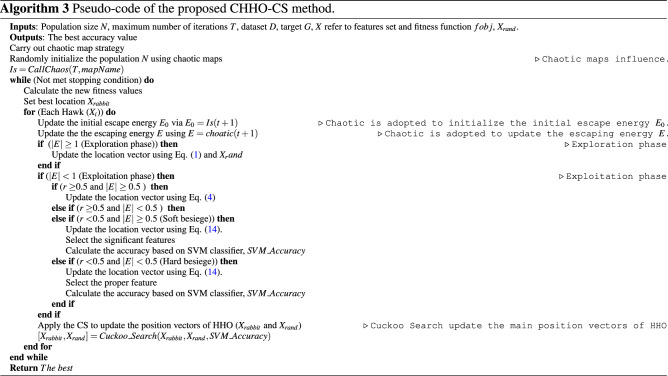

### Feature selection

FS is a data pre-processing step, which is used in combination with the ML techniques. FS permits the selection of a subset without redundancies and desired data. FS can effectively increase the learning accuracy and classification performance. Therefore, the prediction accuracy and data understanding in ML techniques can be improved by selecting the features that are highly correlated with other features. Two features show perfect correlation; however, only one feature is introduced to sufficiently describe the data. Therefore, classification is considered to be a major task in the ML techniques; in classification, data are classified into groups depending on the information obtained with respect to different features. Large search spaces are a major challenge associated with FS; therefore, different MAs are used to perform this task.

### Fitness function

Each candidate solution is evaluated along with the number of iterations to verify the performance of the proposed algorithm. Meanwhile, in classification, the dataset needs to be divided into training and test sets. The fitness function of the proposed CHHO–CS method is defined by the following equation:

15 $$\begin{aligned} Fitness \;function\; (fobj) =\alpha +\beta \frac{|R|}{|C|}-G. \end{aligned}$$

and

16 $$\begin{aligned} Fitness>T \end{aligned}$$

where *R* refers to the classification error and *C* is the total number features for a given dataset *D*. $\beta$ refer to the subset length and $\alpha$ represents the classification performance defined in the range [0, 1]. *T* is a necessary condition and *G* is a group column for the specific classifier. Each step in the algorithm is compared with *T*, where the obtained fitness value must be greater than in order to maximize the solution. It is important to remark that the fitness (or objective) function in Eq. (15) is also used by the CS to compute the the positions of $X_{rand}$ and $X_{rabbit}$.

## Results

To perform the experiments and comparisons, it is necessary to set up the initial values of the problem. In this way, the number of search agents is 30, the problem dimensions 1,665 for the first dataset, and 41 for the second dataset. Meanwhile, the number of iterations is set to 100 and 1,000, number experiments (runs) 30, $\alpha$ is the fitness function 0.99, $\beta$ in the fitness function 0.01, lower bound 0 and upper bound 1. For comparative purposes, seven meta-heuristics algorithms including the standard Cuckoo Search (CS) and Harris Hawks Optimizer (HHO), also ten chaotic maps to verify which of them provides better results are used to verify the proposed method but due to the lack of space we have added the results of the best map only. The selected meta-heuristics and the proposal have the same elements in the population and all of them are randomly initialized. The internal parameters for all the algorithms are provided in Table 2.

**Table 2:** Parameters setting of competitor algorithms used in the comparison and evaluation.

Methods	Parameters
PSO	Agents number = 50
	Velocity = 65
MFO	Agents number = 50
	B = 1
GWO	Agents number = 50
	Number domination = 100
SSA	Agents number = 50
	L = 2 and C = rand
SCA	Agents number = 50
	A = 2
HHO	Agents number = 50
	$E_0$ variable change from $-\,1$ to 1 (Default)
	Beta = 1.5
CS	Agents number = 50
	Discovery rate of align eggs solution = 0.25
	Levy distribution parameter = 1.5
	Step length = 0.01
HHO–CS	Both HHO and CS parameters
CHHO–CS	Both HHO and CS parameters
	$x_0$ = rand default for maps

A common machine learning classifier has been used in experiments including called SVM also was combined with the proposed CHHO–CS method for the classification purpose.

### Performance analysis using UCI datasets

Description and pre-processing of the datasets, results, and comparison of the proposed CHHO–CS is described in the following subsections.

#### UCI Data description

The proposed algorithm is examined on ten benchmark datasets obtained from the UCI machine learning repository^59^ illustrated in Fig. 3 and it is available at “https://www.openml.org/search”.

**Table 3:** Description of the UCI machine learning repository datasets.

No	Dataset	Instances	No features	Classes
D1	Breast cancer	669	9	2
D2	KCL	2,110	21	2
D3	WineEW	178	13	3
D4	WDBC	569	30	2
D5	Lung Cancer	226	23	2
D6	Diabetic	1,151	19	2
D7	Stock	950	9	2
D8	Scene	2,407	299	2
D9	Lymphography	148	18	4
D10	Parkinsons	195	22	2

#### Statistical results

SVM is used for the classification task. Following the previous methodology, in this experiment, iterations are set to 1,000 for each of the 30 runs. The experimental results are reported in Tables 4 and 5. In this experiment, the CHHO–CS-Piece based on SVM achieves the best mean and Std.

**Table 4:** Values of the statistical measures obtained by the competitor algorithms using the SVM classifier with 1,000 iterations over D1, D2, D3, D4 and D5.

Dataset	Methods	Mean	Std	Best	Worst
D1	PSO	8.79E+01	7.80E−01	85.587	84.972
	MFO	8.85E+01	77.70E−01	87.985	87.481
	GWO	8.37E+01	7.90E−01	87.503	87.399
	SSA	8.55E+01	7.85E−01	86.301	85.930
	SCA	8.75E+01	7.70E−01	85.602	85.099
	HHO	8.95E+01	7.55E−01	87.501	86.430
	CS	8.90E+01	7.90E−01	82.503	82.399
	HHO–CS	9.80E+01	7.66E−01	90.102	89.890
	CHHO–CS-Piece	9.89E+01	7.20E−01	91.202	90.591
D2	PSO	8.79E+01	7.80E−01	84.087	83.872
	MFO	8.85E+01	7.70E−01	88.097	87.881
	GWO	8.37E+01	7.90E−01	86.103	86.099
	SSA	8.55E+01	7.85E−01	88.101	87.930
	SCA	8.75E+01	7. 70E−01	87.402	86.909
	HHO	8.95E+01	7.55E−01	89.501	88.430
	CS	8.90E+01	7.95E−01	82.000	81.469
	HHO–CS	8.80E+01	7.66E−01	91.292	91.199
	CHHO–CS-Piece	9.89E+01	7.19E−01	91.502	91.299
D3	PSO	8.79E+01	7.82E−01	85.187	85.179
	MFO	8.85E+01	7.75E−01	87.197	86.980
	GWO	8.37E+01	7.90E−011	86.103	86.999
	SSA	8.55E+01	7.85E−01	87.301	87.131
	SCA	8.75E+01	7. 74E−011	87.112	86.909
	HHO	8.75E+01	7.70E−01	90.001	89.230
	CS	8.90E+011	7.95E−01	82.000	81.869
	HHO–CS	8.80E+01	7.66E−01	90.992	91.999
	CHHO–CS-Piece	8.97E+01	7.11E−01	91.002	90.299
D4	PSO	8.70E+01	7.82E−01	85.187	84.970
	MFO	8.80E+01	7.73E−01	86.177	85.780
	GWO	8.33E+01	7.91E−01	87.121	86.980
	SSA	8.50E+01	7.85E−01	88.103	87.930
	SCA	8.72E+01	7. 73E−01	87.122	86.660
	HHO	8.86E+01	7.56E−01	90.551	89.990
	CS	8.77E+01	7.92E−01	82.312	81.960
	HHO–CS	8.89E+01	7.66E−01	91.991	90.980
	CHHO–CS-Piece	9.09E+01	7.76E−01	92.113	91.950
D5	PSO	8.70E+01	7.88E−01	87.180	86.920
	MFO	8.81E+01	7.75E−01	87.377	86.980
	GWO	8.30E+01	7.93E−01	87.121	86.980
	SSA	8.50E+01	7.80E−01	87.910	87.310
	SCA	8.70E+01	7. 75E−01	92.910	91.560
	HHO	8.90E+01	7.85E−01	92.510	91.410
	CS	8.99E+01	7.80E−01	84.01	83.900
	HHO–CS	8.96E+01	7.76E−01	92.990	91.990
	CHHO–CS-Piece	9.89E+01	7.06E−01	93.801	92.990

**Table 5:** Values of the statistical measures obtained by the competitor algorithms using the SVM classifier with 1,000 iterations over D6, D7, D8, D9 and D10.

Dataset	Methods	Mean	Std	Best	Worst
D6	PSO	8.73E+01	7.82E−01	87.160	86.500
	MFO	8.80E+01	7.72E−01	91.100	91.120
	GWO	8.36E+01	7.90E−01	90.012	88.691
	SSA	8.55E+01	7.80E−01	89.120	88.900
	SCA	8.70E+01	7. 70E−01	87.530	87.091
	HHO	8.85E+01	7.55E−01	90.910	90.769
	CS	8.80E+01	7.70E−01	84.000	83.599
	HHO–CS	8.90E+01	7.66E−01	91.780	90.890
	CHHO–CS-Piece	9.11E+01	7.02E−01	91.590	90.180
D7	PSO	8.29E+01	7.53E−01	82.120	81.920
	MFO	8.39E+01	7.69E−01	87.100	86.431
	GWO	8.30E+01	7.81E−01	84.100	83.771
	SSA	8.29E+01	7.89E−01	82.991	80.190
	SCA	8.13E+01	7.90E−01	84.012	83.060
	HHO	8.49E+01	7.13E−01	85.101	82.920
	CS	8.66E+01	7.30E−01	82.191	81.090
	HHO–CS	8.65E+01	7.17E−01	86.021	85.431
	CHHO–CS-Piece	8.79E+01	7.02E−01	87.709	85.310
D8	PSO	8.29E+01	7.53E−01	82.120	81.920
	MFO	8.32E+01	7.66E−01	87.070	86.530
	GWO	8.33E+01	7.82E−01	84.010	83.570
	SSA	7.83E−01	82.930	82.930	81.990
	SCA	8.13E+01	7. 80E−01	84.011	83.261
	HHO	8.42E+01	7.19E−01	85.011	84.901
	CS	8.52E+01	7.29E−01	82.090	81.199
	HHO–CS	8.55E+01	7.14E−01	86.020	85.730
	CHHO–CS-Piece	8.77E+01	7.01E−01	87.507	86.610
D9	PSO	8.28E+01	7.75E−01	87.190	87.070
	MFO	8.23E+01	7.70E−01	87.020	86.980
	GWO	8.28E+01	7.79E−01	90.502	89.920
	SSA	8.40E+01	7.83E−01	91.502	90.091
	SCA	8.44E+01	7. 92E−01	91.990	90.861
	HHO	8.80E+01	7.45E−01	90.041	89.919
	CS	8.21E+01	7.89E−01	84.090	83.990
	HHO–CS	8.86E+01	7.10E−01	90.821	89.931
	CHHO–CS-Gauss	8.82E+01	7.02E−01	93.639	92.470
D10	PSO	8.24E+01	7.79E−01	79.180	78.471
	MFO	8.25E+01	7.78E−01	80.120	79.080
	GWO	8.26E+01	7.79E−01	80.001	79.022
	SSA	8.43E+01	7.89E−01	80.102	80.090
	SCA	8.47E+01	7. 94E−01	80.891	79.360
	HHO	8.82E+01	7.35E−01	81.090	80.910
	CS	8.24E+01	7.80E−01	878.091	76.091
	HHO–CS	8.88E+01	7.30E−01	80.991	80.230
	CHHO–CS-Piece	8.81E+01	7.09E−01	82.019	80.012

#### Classification results

Since SVM is one of the most promising methods of classification, its performance needs to be analyzed. In this experiment, the number of iterations are set to 1,000, also the obtained results are reported in Tables 6 and 7. Notably, the CHHO–CS-Piece based on SVM obtains the best classification accuracy, sensitivity, specificity, recall, precision, and F-measure.

**Table 6:** Classification values obtained by the competitor algorithms using the SVM classifier with 1,000 iterations over D1, D2, D3, D4 and D5.

Dataset	Methods	Accuracy	Sensitivity	Specificity	Recall	Precision	F-measure
D1	PSO	85.587	32.800	46.100	32.800	54.430	40.950
	MFO	87.985	33.150	47.450	33.150	54.990	41.750
	GWO	87.503	33.100	47.150	33.100	55.150	41.710
	SSA	86.301	33.150	47.120	33.150	54.190	41.540
	SCA	85.602	31.990	46.350	31.990	54.550	40.570
	HHO	88.709	33.250	47.700	33.250	54.490	41.420
	CS	84.003	31.510	45.300	31.510	54.690	40.760
	HHO–CS	90.102	33.950	48.930	33.950	56.570	41.910
	CHHO–CS-Piece	91.202	33.590	48.950	33.590	55.330	42.590
D2	PSO	84.087	30.851	47.420	30.851	54.740	41.940
	MFO	88.097	32.151	48.426	32.151	55.150	40.847
	GWO	86.103	31.551	47.906	31.551	54.945	41.940
	SSA	88.101	31.950	48.920	31.950	55.240	41.980
	SCA	87.402	31.350	48.120	31.350	54.940	40.540
	HHO	89.501	32.150	48.920	32.150	55.750	41.240
	CS	82.000	29.950	47.420	29.950	51.955	40.640
	HHO–CS	91.292	33.150	49.120	33.150	56.940	41.647
	CHHO–CS-Piece	91.502	33.250	47.250	33.250	55.950	41.840
D3	PSO	85.187	30.851	47.920	30.851	54.745	40.940
	MFO	87.197	30.961	48.420	30.961	55.145	41.347
	GWO	86.103	30.450	48.150	30.450	55.045	41.150
	SSA	87.301	30.650	47.450	30.650	55.145	41.350
	SCA	87.102	30.750	47.410	30.750	54.950	41.370
	HHO	90.001	32.450	49.120	32.450	56.140	42.940
	CS	82.000	30.150	45.120	30.150	52.145	39.940
	HHO–CS	90.992	33.551	49.250	33.551	54.340	40.947
	CHHO–CS-Piece	91.002	33.750	49.750	33.750	54.600	41.240
D4	PSO	85.187	30.950	47.936	30.950	54.640	40.247
	MFO	86.177	31.100	48.150	31.100	54.950	40.807
	GWO	87.121	31.250	48.540	31.250	55.140	41.240
	SSA	88.103	31.300	48.860	31.300	55.250	41.740
	SCA	87.122	31.100	48.156	31.100	54.145	40.940
	HHO	90.551	32.150	49.960	32.150	55.640	42.940
	CS	82.312	29.750	46.520	29.750	53.140	39.640
	HHO–CS	91.991	32.350	49.120	32.350	55.740	42.870
	CHHO–CS-Piece	92.113	32.890	49.996	32.890	55.995	42.970
D5	PSO	87.180	31.710	48.240	31.710	55.200	43.940
	MFO	87.377	30.200	48.220	30.150	54.250	41.970
	GWO	87.121	31.650	47.160	31.650	54.950	41.250
	SSA	87.910	31.700	48.720	31.700	55.850	43.280
	SCA	92.910	32.300	48.100	31.200	55.730	42.140
	HHO	92.510	32.350	48.710	32.350	55.350	43.990
	CS	84.010	30.100	47.220	30.100	53.451	40.150
	HHO–CS	92.990	33.160	49.740	33.160	56.255	44.870
	CHHO–CS-Piece	93.801	33.250	49.190	33.250	56.850	44.590

**Table 7:** Classification values obtained by the competitor algorithms using the SVM classifier with 1,000 iterations over D6, D7, D8, D9 and D10.

Dataset	Methods	Accuracy	Sensitivity	Specificity	Recall	Precision	F-measure
D6	PSO	87.160	30.280	48.490	30.280	55.560	43.890
	MFO	91.100	30.390	48.770	30.390	55.100	43.893
	GWO	90.012	30.299	47.790	30.299	54.740	43.471
	SSA	89.120	30.650	48.550	30.120	54.999	43.595
	SCA	87.530	31.996	48.290	31.996	55.470	442.25
	HHO	90.910	32.895	48.990	32.895	55.994	44.397
	CS	82.312	29.750	46.520	29.750	53.140	39.640
	HHO–CS	91.780	32.766	49.990	32.766	56.492	44.992
	CHHO–CS-Piece	91.590	33.252	49.660	33.252	56.991	44.899
D7	PSO	82.120	31.901	48.742	31.901	55.732	43.902
	MFO	87.100	30.901	48.629	30.901	54.753	43.991
	GWO	84.100	31.989	47.979	31.989	54.933	43.962
	SSA	82.991	31.969	48.820	31.969	55.939	43.599
	SCA	84.012	31.359	48.990	31.359	55.960	42.951
	HHO	85.101	32.298	48.980	32.298	55.599	44.992
	CS	82.191	31.849	47.359	31.540	53.859	40.932
	HHO–CS	86.021	31.391	49.377	31.391	56.990	44.993
	CHHO–CS-Piece	87.709	31.102	49.291	31.102	55.852	44.711
D8	PSO	82.120	31.979	48.472	31.979	55.339	43.920
	MFO	87.070	30.192	48.732	30.192	54.852	43.909
	GWO	84.010	31.289	47.772	31.289	54.931	43.269
	SSA	82.930	31.990	48.830	31.990	55.901	43.893
	SCA	84.011	31.952	48.929	31.952	55.968	42.952
	HHO	85.011	32.297	48.987	32.297	55.799	44.399
	CS	82.090	31.537	47.452	31.537	53.955	40.956
	HHO–CS	86.020	31.991	49.971	31.991	56.599	44.930
	CHHO–CS-Piece	87.507	31.010	49.091	31.010	55.950	44.410
D9	PSO	87.190	31.909	48.970	31.909	55.910	43.919
	MFO	87.020	30.902	48.970	30.902	54.920	43.991
	GWO	90.502	31.990	47.979	31.990	54.933	43.962
	SSA	82.991	31.969	48.820	31.969	55.939	43.492
	SCA	84.012	31.359	48.990	31.359	55.960	42.951
	HHO	85.101	32.298	48.980	32.298	55.599	44.992
	CS	82.191	31.849	47.359	31.540	53.859	40.932
	HHO–CS	86.021	31.391	49.377	31.391	56.990	44.993
	CHHO–CS-Piece	87.709	31.102	49.291	31.102	55.852	44.711
D10	PSO	82.120	31.979	48.472	31.979	55.339	43.920
	MFO	87.070	30.192	48.732	30.192	54.852	43.909
	GWO	84.010	31.289	47.772	31.289	54.931	43.269
	SSA	82.930	31.990	48.830	31.990	55.901	43.893
	SCA	84.011	31.952	48.929	31.952	55.968	42.952
	HHO	85.011	32.297	48.987	32.297	55.799	44.399
	CS	82.090	31.537	47.452	31.537	53.955	40.956
	HHO–CS	86.020	31.991	49.971	31.991	56.599	44.930
	CHHO–CS-Piece	87.507	31.010	49.091	31.010	55.950	44.410

### Performance analysis using chemical datasets

#### Description of chemical datasets

In this study, two different datasets are used to experimentally evaluate the performance of the proposed method. (1) The MAO dataset comprises 68 molecules and is divided into two classes: 38 molecules that inhibit MAO (antidepressants) and 30 molecules that do not. MAO is available at http://iapr-tc15.greyc.fr/links.html. Each molecule should have a mean size of 18.4 atoms, and the mean degree of the atoms is 2.1 edges. In addition, the smallest molecule contains 11 atoms, whereas the largest one contains 27 atoms; each molecule has 1,665 descriptors. (2) The QSAR biodegradation dataset comprises 1,055 chemical compounds, 41 molecular descriptors, and one class; it is available at http://archive.ics.uci.edu/ml/datasets/QSAR+biodegradation. These chemical compounds are obtained from the National Institute of Technology and Evaluation of Japan (NITE). The MAO dataset is transformed into a line notation form to describe the structure of the simplified molecular-input line-entry system (SMILES) using the open babel software^60^; E-dragon^61^ is subsequently applied to obtain the molecular descriptor. Information obtained with respect to the second QSAR biodegradation dataset was preprocessed by the Milano Chemometrics and QSAR Research Group, University of Milano-Bicocca and is available at http://www.michem.unimib.it/

#### Data preprocessing

**Figure 4:**
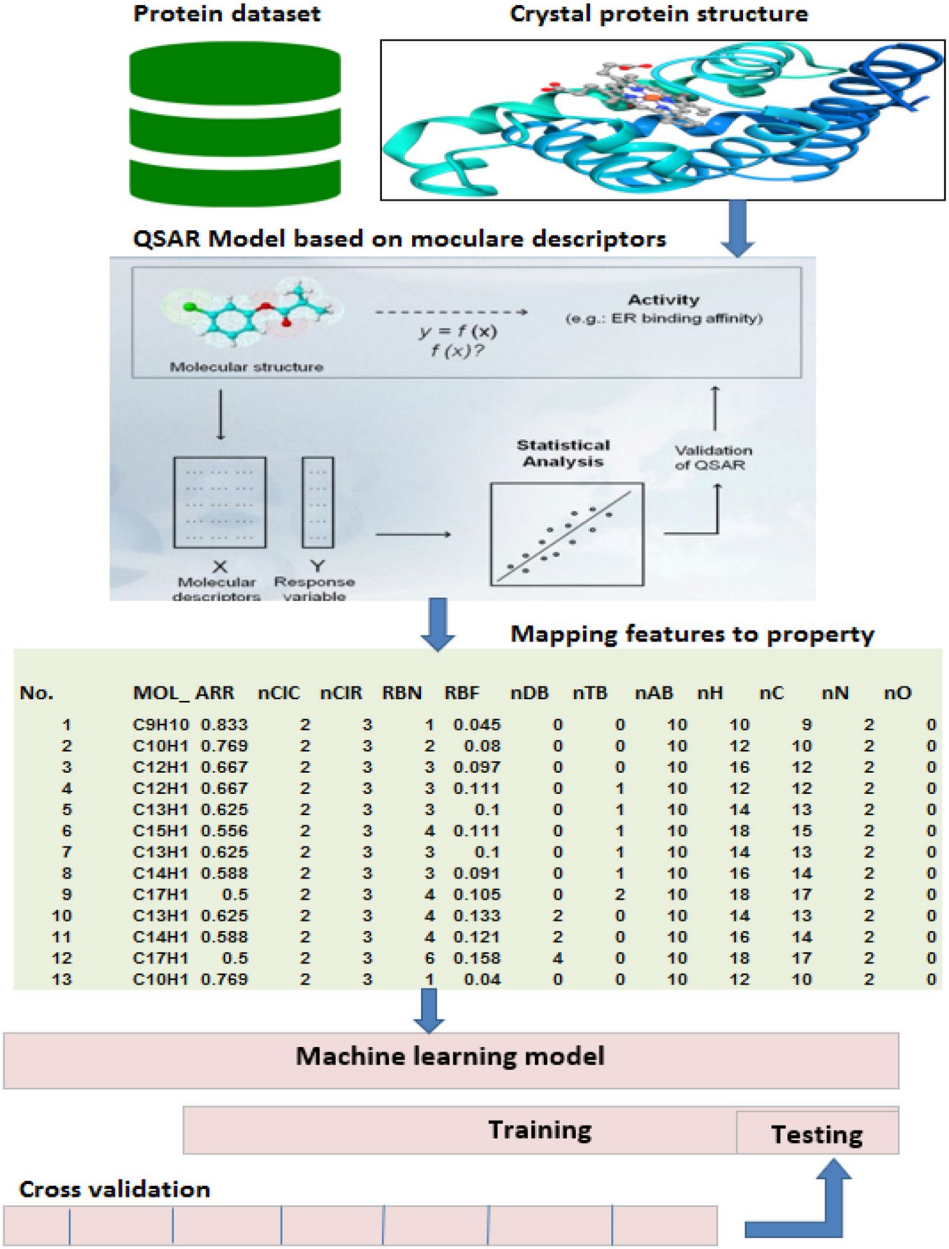
Mapping from a molecular to a space of features.

Here, the required steps to preprocess the data set information are presented. The information obtained from the molecules is transferred to the features representing chemical compounds^36,39^. The data obtained from the proteins are stored in a special chemical format. Further, the software should be used to transfer the information into the isomeric SMILES. The data set contains different instances with specific multidimensional attributes (commonly two-dimensional 2*D* and 3*D* according to the QSAR model. The E-dragon software is used to compute the descriptors from this dataset. The descriptors contain physicochemical or structural information as solvation properties, molecular weight, aromaticity, volume, rotatable bonds, molecular walk counts, atom distribution, distances, interatomic, electronegativity, and atom types. They are used for determining values of generations and instances which belong to a class as shown in Fig. 4.

#### Statistical results

Here, the SVM is used for the classification task. Following the previous methodology, in the first experiment, iterations are set to 100 for each of the 30 runs. The experimental results are reported in Tables 8. In this experiment, the CHHO–CS-Piece based on SVM obtains the best mean and Std. The same rank is obtained for maximizing the classification accuracy solution, Sensitivity, Specificity, Recall, Precision, and F measure. In this case, the HHO–CS with SVM is the second-ranked in mean value, Std, and maximizing the classification accuracy solution, sensitivity, specificity, recall, precision, and F-measure. The iterations are configured to 1,000; the idea is to obtain the best solutions. In this case, the results are presented in Table 9, where the CHHO–CS-Piece combined with the SVM is the fist ranked approach for the mean value, and Std, the same occurs for maximizing the classification accuracy solution, sensitivity, specificity, recall, precision, and F-measure. Meanwhile, the second algorithm in the rank is the HHO–CS with SVM for mean value, Std, and maximizing the classification accuracy solution.

**Table 8:** Values of the statistical measures obtained by the competitor algorithms using the SVM classifier with 100 iterations.

Dataset	Methods	Mean	Std	Best	Worst
MAO	PSO	8.07E+01	7.30E−01	87.987	86.472
	MFO	8.83E+01	7.36E−01	85.285	84.981
	GWO	8.20E+01	7.40E−01	85.003	84.999
	SSA	8.40E+01	7.32E−01	87.501	87.430
	SCA	8.60E+01	7.33E−01	86.002	85.699
	HHO	9.50E−01	7.45E−02	94.247	93.011
	CS	8.50E−01	2.60E−01	84.232	83.178
	HHO–CS	9.60E−01	7.32E−02	95.320	94.334
	CHHO–CS-Piece	**9.76E−01**	7.15E−02	96.180	95.702
QSAR	PSO	8.70E+01	7.30E−01	79.987	79.472
	MFO	8.30E+01	7.10E−01	80.285	80.981
	GWO	8.40E+01	7.04E−01	80.503	80.399
	SSA	8.60E+01	7.35E−01	79.501	78.430
	SCA	8.50E+01	7.06E−01	80.002	79.999
	HHO	8.19E−01	6.69E−03	80.990	81.017
	CS	8.17E−01	6.71E−04	78.902	79.011
	HHO–CS	8.28E−01	6.66E−04	81.970	82.011
	CHHO–CS-Piece	**8.33E−01**	6.68E−04	82.521	82.711

**Table 9:** Values of the statistical measures obtained by the competitor algorithms using the SVM classifier with 1,000 iterations.

Dataset	Methods	Mean	Std	Best	Worst
MAO	PSO	8.15E+01	7.22E+00	87.981	86.981
	MFO	8.12E+01	0.00E+00	87.176	86.176
	GWO	9.25E+01	7.20E−01	90.705	89.705
	SSA	9.12E+01	7.17E−01	92.647	91.235
	SCA	9.12E+01	7.17E−02	92.647	91.176
	HHO	9.55E−01	7.48E−02	95.259	94.061
	CS	8.55E−01	2.90E−01	84.300	83.523
	HHO–CS	9.60E−01	7.40E−02	95.530	95.440
	CHHO–CS-Piece	**9.85E−01**	7.23E−02	96.190	95.950
QSAR	PSO	8.47E+01	7.30E−01	79.887	79.472
	MFO	8.33E+01	7.16E−01	80.985	80.681
	GWO	8.40E+01	7.94E−01	80.603	80.499
	SSA	7.40E+01	7.05E−01	78.801	78.630
	SCA	8.42E+01	7.16E−01	80.002	79.999
	HHO	8.39E−01	1.41E−03	80.971	81.210
	CS	8.28E−01	2.42E−02	79.800	79.901
	HHO–CS	8.40E−01	1.40E−03	82.301	82.511
	CHHO–CS-Piece	**8.42E−01**	1.39E−03	84.012	84.001

#### Classification results

Since SVM is one of the most promising methods of classification, its performance needs to be analyzed. In the first experiment, iterations are set to 100; the experimental results are reported in Table 10. In this experiment, the CHHO–CS-Piece based on SVM obtains the best results. In this case, the HHO–CS with SVM is the second-ranked in most of the assessment criteria. A final experiment for SVM is performed by using 1,000 iterations and the reported values in Table 11 confirms that the CHHO–CS-Piece combined with the SVM is the first ranked approach. Meanwhile, HHO–CS with SVM is the second-ranked algorithm in most of the assessment criteria.

**Table 10:** Classification values obtained by the competitor algorithms using the SVM classifier with 100 iterations.

Dataset	Methods	Accuracy	Sensitivity	Specificity	Recall	Precision	F-measure
MAO	PSO	87.987	33	33.890	49.950	56.740	42.901
	MFO	85.285	33.930	50.150	33.930	56.9507	43.201
	GWO	85.003	34.100	50.200	34.100	57.150	43.901
	SSA	87.501	34.250	50.250	34.250	57.400	44.101
	SCA	86.002	34.400	50.700	34.400	57.530	44.501
	HHO	94.247	49.930	64.160	49.930	66.536	55.130
	CS	84.232	33.650	49.920	33.650	56.540	42.851
	HHO–CS	95.320	50.120	67.816	50.120	68.392	59.646
	CHHO–CS-Piece	**96**.**180**	53.941	71.660	53.941	73.625	62.540
QSAR	PSO	79.987	49.610	66.950	49.610	68.190	58.950
	MFO	80.285	49.750	66.980	49.750	68.250	59.100
	GWO	80.503	49.800	67.130	49.800	68.300	59.150
	SSA	79.501	49.600	67.300	49.600	68.200	59.300
	SCA	80.002	49.750	67.350	49.750	68.150	59.450
	HHO	81.070	49.720	67.710	49.720	66.536	58.950
	CS	79.001	49.510	66.920	49.510	68.592	58.851
	HHO–CS	82.170	49.820	67.816	49.820	68.690	58.640
	CHHO–CS-Piece	**82**.**720**	49.540	67.460	49.540	68.590	62.540

**Table 11:** Classification values obtained by the competitor algorithms using the SVM classifier with 1,000 iterations.

Dataset	Methods	Accuracy	Sensitivity	Specificity	Recall	Precision	F-measure
MAO	PSO	87.981	40.540	50.120	40.540	56.740	45.360
	MFO	87.176	40.750	50.520	40.750	56.950	45.470
	GWO	90.705	41.150	50.720	41.150	57.150	45.800
	SSA	92.647	41.350	50.830	41.350	57.400	45.900
	SCA	92.647	41.450	50.850	41.450	57.530	46.100
	HHO	95.259	51.331	66.043	51.331	69.024	58.172
	CS	84.300	40.342	50.021	40.342	60.990	45.062
	HHO–CS	95.530	53.444	69.830	53.444	71.930	62.846
	CHHO–CS-Piece	**96**.**190**	55.485	73.843	55.485	75.727	66.182
QSAR	PSO	79.887	40.540	50.100	40.540	61.190	45.160
	MFO	80.985	40.650	50.150	40.650	61.200	45.190
	GWO	80.603	40.710	50.250	40.710	61.150	45.490
	SSA	78.801	40.820	50.300	40.820	61.090	45.510
	SCA	80.002	40.930	50.530	40.930	61.100	45.550
	HHO	81.201	51.940	69.043	51.940	70.920	64.950
	CS	79.901	45.940	55.021	45.940	69.990	65.162
	HHO–CS	82.501	52.420	69.130	52.420	71.130	65.150
	CHHO–CS-Piece	**84**.**001**	52.540	69.340	52.540	71.870	65.880

### The convergence analysis

**Figure 5:**
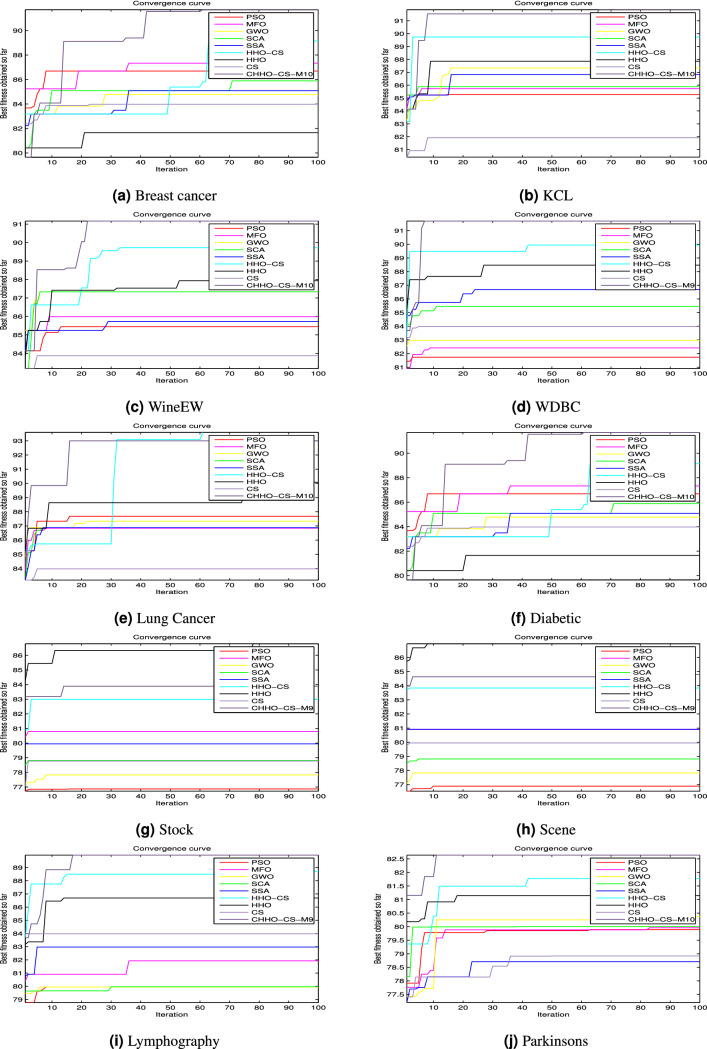
Convergence curves for the best CHHO–CS-based chaotic map and the competitor algorithms using SVM on ten UCI datasets with 100 iterations.

**Figure 6:**
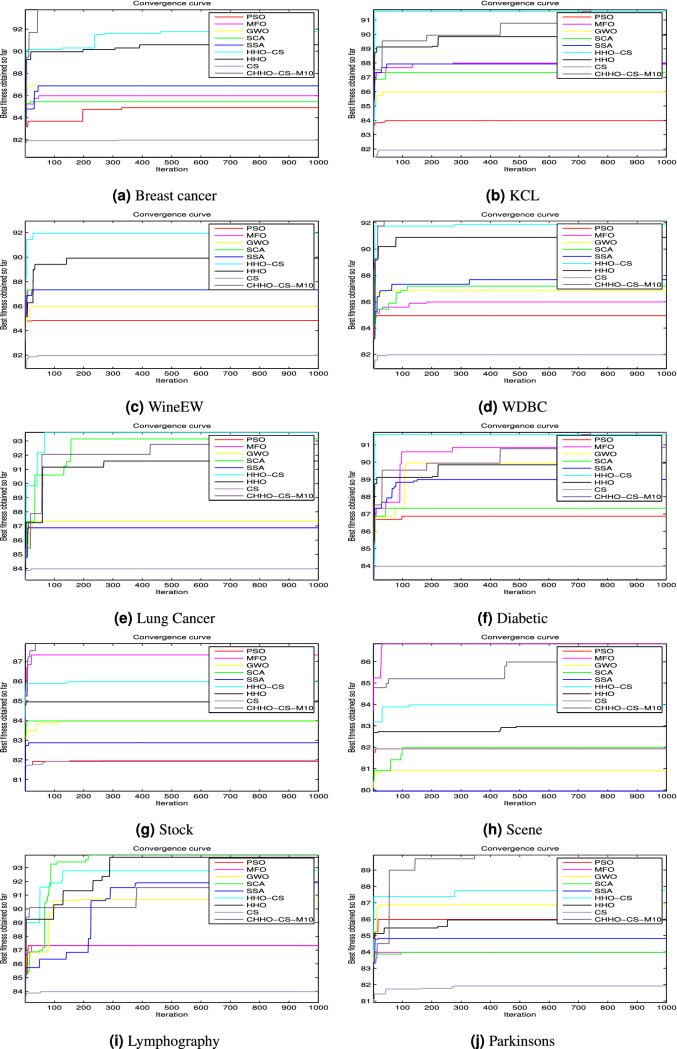
Convergence curves for the best CHHO–CS-based chaotic map and the competitor algorithms using SVM on ten UCI datasets with 1,000 iterations.

This section aims to analyze the convergence of the proposed CHHO–CS based chaotic maps presented in this paper. Figures 5 and 6 shows the convergence curves for the competitor algorithms over the ten UCI Machine Learning Repository datasets along the iterative process 100, and 1,000 iterations respectively. Over the ten UCI datasets, the convergence curves plotted in Figs. 5 and 6 provides evidence that the proposed CHHO–CS method using SVM obtained the best results compared with the original HHO and CS algorithms and the other competitor algorithms along with the two-stop criteria (100 and 1,000 iterations).

**Figure 7:**
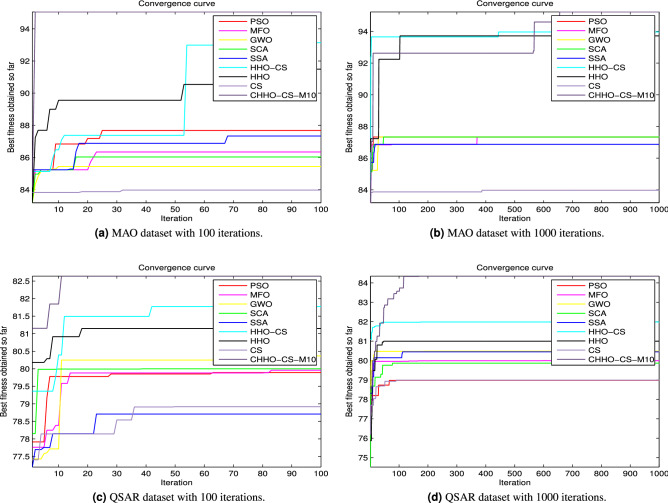
Convergence curves for the best CHHO–CS-based chaotic map and the competitor algorithms using SVM on MonoAmine Oxidase (MAO) and QSAR Biodegradation datasets. (**a**,**b**) MAO dataset with 100, and 1,000 iterations respectively. On the other hand, (**c**,**d**) QSAR biodegradation dataset with 100, and 1,000 iterations respectively.

On the other hand, the convergence curves plotted in Fig. 7a–d provide evidence that the proposed CHHO–CS method with SVM classifier obtained over the two datasets (MAO and QSAR biodegradation) the best results compared with the original HHO and CS algorithms and the other competitor algorithms along with the two-stop criteria (100 and 1,000 iterations).

### Discussion

According to the aforementioned results for both of the UCI datasets and the two chemical datasets (MonoAmine Oxidase (MAO) and QSAR biodegradation datasets), the CHHO–CS maximizes the accuracy and reduces the number of selected features. Also, the obtained Std values are increasing directly when the number of iterations increases for the proposed CHHO–CS method with the SVM classifier. The statistic metrics as mean, Std, best, and worst, as well as the classification assessment, indicate that chaotic maps introduce better results in comparison with the standard approaches. The evidence of this fact can be observed in the convergence curves as shown in Figs. 5, 6 and 7, where the CHHO–CS method based chaotic map with SVM is applied over the UCI datasets and the two chemical datasets (MOA and QSAR).

In worthwhile, the convergence curve is presented because it is a graphical form to study the relationship between the number of iterations and the fitness function. It declares the best-performed algorithm by comparison between various approaches and when increasing the number of iterations, it represents a direct correlation. The convergence curves plotted in Fig. 5a–j revealed that the proposed CHHO–CS-Piece method achieved better results compared with the competitor algorithms. Also, in the same context, the convergence curves plotted in Fig. 6a–j revealed that the proposed CHHO–CS-Piece method achieved better results compared with the competitor algorithms.

To sum up, the experiments were conducted on MOA and QSAR biodegradation datasets and the obtained results are interesting and due to the lack of space, we have added the results of the best map only. For example, in the first MOA dataset with the SVM classification technique in different stop conditions 100, and 1,000 iterations as shown in Fig. 7a–d, respectively. Moreover, on the MAO dataset, with 100 and 1,000 iterations, it is interesting that CHHO–CS-Piece with SVM is better than the other competitor algorithms. Meanwhile, for the second QSAR biodegradation dataset, the optimal solutions with SVM are computed with 100, and 1,000 iterations as stop condition, it is interesting that the version CHHO–CS-Piece with SVM provides the optimal solutions in comparison with the other metaheuristic algorithms.

## Conclusion

metaheuristic algorithms and machine learning techniques are important tools that can solve complex tasks in the field of cheminformatics. The capabilities of MAs and ML to optimize and classify information are useful in drug design. However, these techniques should be highly accurate to obtain optimal compounds. In this paper, a hybrid metaheuristic method termed CHHO–CS which combined the Harris hawks optimizer (HHO) with operators of the cuckoo search (CS) and chaotic maps (C) in order to enhance the performance of the original HHO. Moreover, the proposed CHHO–CS method was combined with the support vector machine (SVM) as machine learning classifiers for conducting the chemical descriptor selection and chemical compound activities. The main tasks of the proposed method are to select the most important features and classify the information in the cheminformatics datasets (e.g., MAO and QSAR biodegradation). The experimental results confirm that the use of chaotic maps enhances the optimization process of the hybrid proposal. It is important to mention that not all the chaotic maps are completely useful, and it is necessary to decide when to use one or another. As expected, this is dependent on the dataset and the objective function. Comparisons of the proposed CHHO–CS method with the standard algorithms revealed that the CHHO–CS yields superior results with respect to cheminformatics using different stop criteria. In the future, the proposed CHHO–CS method can be used as a multi-objective global optimization or feature selection paradigm for high-dimensional problems containing many instances to increase the classification rate and decrease the selection ratio of attributes.
